# Resectability and prognosis of gallbladder cancer: a cross-sectional study of 100 cases from a tertiary care centre of Eastern Nepal

**DOI:** 10.1097/MS9.0000000000000699

**Published:** 2023-04-17

**Authors:** Narendra Pandit, Durga Neupane, Dinesh Nalbo, Sameer Bhattarai, Kunal B. Deo, Lokesh S. Jaiswal, Shailesh Adhikary

**Affiliations:** a Department of Surgical Gastroenterology, B. P Koirala Institute of Health Sciences (BPKIHS), Dharan; b Department of Surgical Gastroenterology, Birat Medical College Teaching Hospital, Biratnagar, Nepal; c Department of Surgery, B. P Koirala Institute of Health Sciences (BPKIHS), Dharan, Nepal; d Department of Surgical Gastroenterology, B. P Koirala Institute of Health Sciences (BPKIHS), Dharan, Nepal

**Keywords:** CA 19-9, extended cholecystectomy, gallbladder carcinoma, median survival, resection

## Abstract

Gallbladder cancer (GBC) is the fifth most common neoplasm of the digestive tract and has an overall incidence of 3 per 100 000 people. Only 15–47% of the preoperatively known GBC are suitable for resection. The objective of the study was to investigate the resectability and prognosis of GBC patients.

**Materials and methods:**

It is a prospective observational study including all cases of primary cancers of the gallbladder in the Department of Surgical Gastroenterology at a tertiary care center over the period from January 2014 to December 2019. The primary endpoint was resectability and overall survival.

**Results:**

During the study period, 100 patients with GBC were reported. The mean age at the time of diagnosis was 52.5 years, with a female predominance (67%). The curative intent resection (radical cholecystectomy) was possible in 30 (30%) patients; while 18 (18%) required palliative surgical treatment. The overall survival of the entire group was 9 months; while those patients who underwent surgery with curative intent had a median overall survival of 28 months after a median follow-up of 42 months.

**Conclusion:**

This study found that only one-third of patients achieve radical surgery with curative intent. Overall, the prognosis of patients is poor with a median survival of less than a year due to the advanced stage disease. Multimodality treatment, screening ultrasound, and neo-/adjuvant therapy may improve survival.

## Introduction

Highlights

- Gallbladder cancer is the fifth most common neoplasm of the digestive tract and has an overall incidence of 3 per 100 000 people.
- Only 15–47% of the preoperatively known gallbladder cancer are suitable for resection.
- This study found that only one-third of patients achieve radical surgery with curative intent.
- Multimodality treatment, screening ultrasound, and neo-/adjuvant therapy may improve survival.

Gallbladder cancer (GBC) is the most common cancer of the biliary tract and has an overall incidence of 3 per 100 000 people^1^. Only 15–47% of the preoperatively known GBCs are suitable for resection^2^. This is attributed to the aggressive tumor biology, thin walled gallbladder, and close approximation of it to the liver, leading to early spread. Additionally, the gallbladder is situated in the ‘busy area’ of the portal triad leading to involvement of the major vascular structures, and hence unresectability. The outcome of GBC is poor with an overall 5-year survival rate of less than 5%. A 5-year survival rate up to 75% can be achieved in early-stage or incidentally detected disease, provided stage-adjusted therapy is performed^3^. On the other hand, if GBC is locally advanced, and radicle resection combined with neo-/adjuvant chemotherapy is subjected, the 5-year overall survival is only 20–30%^3^.

With reference to GBC cancer statistics (2018), Nepal ranks the fifth highest incidence rate in the world, with 6.7 cases/100 000 population per annum^4^,^5^. Our center caters 2.5 million populations, and we frequently observe cases of GBC in the outpatient clinic. The aim of our study was to evaluate the incidence of resection of GBC with curative intent and the overall survival. This cross-sectional study has been reported according to strengthening the reporting of cohort, cross-sectional and case-control studies in surgery (STROCSS) guideline^6^.

## Materials and methods

After Institutional Review Board approval (10.082), a review of a prospectively collected database of all patients with GBC treated at our tertiary academic hospital between January 2014 and December 2019 was done. Data collected included patient demographics, incidental versus nonincidental GBC, presence of gallstones, symptoms at presentation, presence of jaundice, stage of the disease, resectability, CA19-9 level, extents of resection, resection (R) status, complications, pathology, recurrence, and survivals. The seventh edition of the American Joint Committee on Cancer staging system was used for pathological and/or clinical staging. Only patients with a histological conformation of gallbladder cancer were included. Patients were excluded if the indication for surgery was xanthogranulomatous cholecystitis or benign disease, or were lost to follow-up. The primary endpoint was the incidence of resectability and median survival of patients after curative intent surgery for GBC.

### Patient management

Preoperative staging routinely included CA 19-9 value determination and abdominal computed tomography (CT). Magnetic resonance cholangio-pancreatography was performed in all resectable jaundiced patients after CT. In jaundiced patients with resectable disease, surgery was scheduled only if adequate FLR hypertrophy was observed. Preoperative biliary drainage was performed for deep jaundice (bilirubin >10 mg/dl), cholangitis, major liver resection, or malnutrition. Surgery was performed whenever complete resection was achievable. Distant metastases, extensive infiltration of the hepatoduodenal ligament, and aorto-caval LN metastases were considered contraindications to surgery. A combined pancreatoduodenectomy was performed in case of infiltration of the duodeno-pancreatic area by the GBC or by large retropancreatic LN metastases. Colonic or gastric resection was performed in case of direct tumor infiltration. The standard scheduled procedure for GBC was wedge resection or bisegmentectomy (segment 4b-5-if large area of liver infiltration by the tumor), en-bloc cholecystectomy, and hepatoduodenal ligament node dissection.

### Adjuvant treatment and follow-up

Postoperative chemotherapy was delivered according to the patient’s performance status and pathological findings. All patients were followed-up every 3 months with a physical examination, CA 19-9 determinations, and abdominal CT. Six patients were lost to follow-up.

### Definitions

A major hepatectomy was defined as the resection of three Couinaud segments. Operative mortality was defined as death within 90 days after surgery or before discharge from the hospital. Morbidity included all postoperative complications and was classified according to the Clavien–Dindo classification.

### Statistical analysis

Continuous variables of demographic data, clinical characteristics, and lab values, were summarized using descriptive statistics and expressed as median (Interquartile Range) for continuous variables and frequency (percentage) for categorical variables. The results were presented in graphical and tabular form. All data were entered in Microsoft Excel and analyzed in Statistical Package for Social Science (SPSS) version 17.0.

## Results

### Patient characteristics

During the study period, 100 patients with GBC were reported. The mean age at the time of diagnosis was 52.5 (40–88) years, with a clear female predominance (67%). The presenting symptoms were dominated by right hypochondrial pain (50%), jaundice (56%), and gastric outlet obstruction (10%). Associated gallstone was demonstrated in 70%. Ten patients were young-onset (<40 years) GBC at presentation. Two patients with obstructive jaundice, but resectable disease underwent preoperative biliary drainage. Table 1 shows the clinical profile and treatment outcome of patients enrolled in the study.

**Table 1:** Clinical profile and treatment outcome of patients enrolled in the study (*n*=100)

Parameters	*N* (%)
Age (mean), years (range)	52.5 (40–88)
Male: Female ratio	33:67
Associated gallstone (%)	70
Obstructive jaundice (%)	56
Gastric outlet obstruction (%)	10
CA 19-9 level (ng/mL) mean (range)	110 (39–1000)
Tumor AJCC stage (%)	
I	3
II	6
III	19
IVa	12
IVb	60
Perioperative morbidity (%)	23
Operative mortality (%)	3
R0 resection, *n* (%)	20 (66.6)
Mean number of lymph nodes harvested, n(range)	4 (0–7)
Recurrence in curative intent, *n* (%)	14 (46.6)
Adjuvant chemotherapy completion, *n* (%)	12 (40)

### Surgery

**Figure 1:**
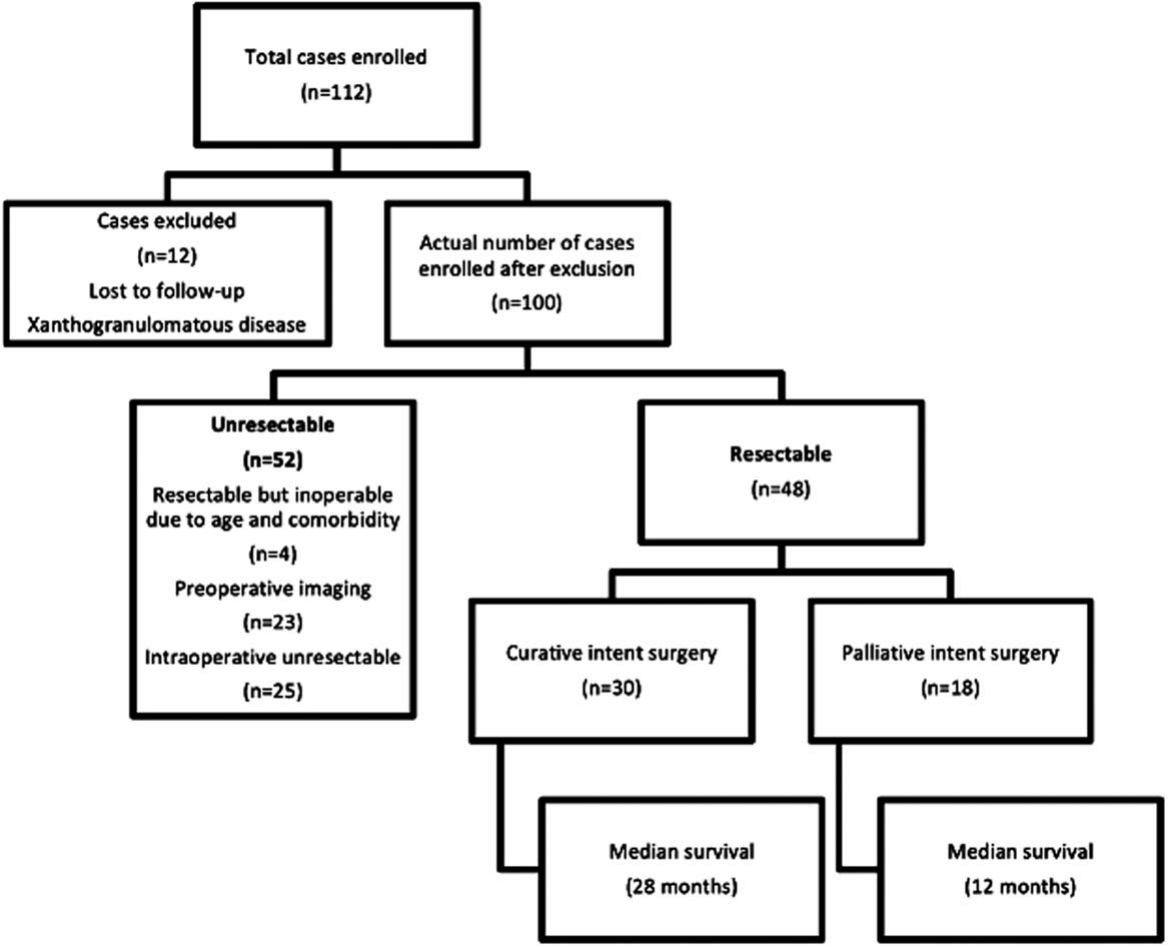
Consort diagram of patients with gallbladder carcinoma, respective surgical intervention, and outcome.

The curative intent resection was possible in 30 (30%) patients; while 18 (18%) required palliative surgical treatment. Metastatic disease was dominant in 48 (48%) patients who were managed with the best palliative treatment. The incidental GBC (IGBC) was observed in 7 (7%) patients. Curative intent surgery was possible in four out of seven patients with IGBC. The remaining IGBC were metastatic due to delayed presentation to our center. Resectable tumor but inoperable disease (because of age and comorbidity) was observed in four patients. Of the 48 patients who were metastatic, 23 were clearly unresectable based on preoperative imaging. Further, 25 patients were found to have unresectable disease at staging laparoscopy/laparotomy. Figure 1 shows the flowchart of patients with GBC and the respective surgical intervention and outcome.

All patients except three had a wedge resection of the liver. An extended right hepatectomy with bile duct resection was performed in two patients; one underwent hepatopancreatoduodenectomy; and one required segmental colectomy.

### Pathology

At histopathological examination, adenocarcinoma was found in 95 patients and squamous cell carcinoma in five patients. After curative intent resection, out of 30 patients, only 12 received and completed adjuvant chemotherapy. R0 resection was achieved in 66.6% (20/30) patients. All except three patients were locally advanced (T3, N1) GBC in the curative intent group. The median number of lymph nodes harvested was 4 (0–7) in extended cholecystectomy. Six patients who underwent extended cholecystectomy for presumed GBC were excluded because of xanthogranulomatous cholecystitis.

### Outcome

**Figure 2:**
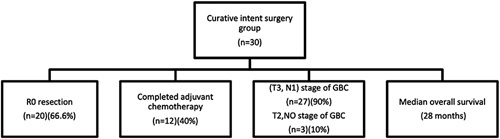
Outcome of patients who underwent curative intent surgery for gallbladder carcinoma.

The perioperative morbidity and mortality was observed in 23% (major: 20%; minor: 80%) and 3%, respectively. The overall survival of the entire group was 9 months; while those patients who underwent surgery with curative intent have median overall survival of 28 months after a median follow-up of 42 months (12–64 months). Tumor recurrence occurred in 46% (14/30) patients (1 in the IGBC group); following curative intent resection. Three patients had a local recurrence only, three patients had metastatic disease and a local recurrence, and eight patients had metastatic disease only. The incidence of resectability was 30% and the median survival of 28 months in the curative intent surgery group (Fig. 2).

## Discussion

GBC is a common malignancy in our part. Unfortunately, the majority (70%) of our patients present with advanced stage disease and are unresectable as compared to other similar studies from the Indian subcontinent^7^,^8^. Only 30% of patients are subjected to curative intent surgery with a median survival of ~28 months. The further findings observed in the present study was high number of IGBC (7 vs. 1%) and young-onset GBC- 10%, compared to similar study in the world.

In the study by Subedi *et al.* from Nepal, the incidence of GBC was higher in the Kathmandu Valley than in the cities of India and China. Cancer of the gallbladder was among the top five commonest and lethal cancers in both sex^9^. In addition to the high incidence of GBC in Nepal, the presentation of patients is usually late with an advanced stage of disease. Some studies have shown an association of history of gallstone, smoking, and early menarche with GBC in Nepal^10^,^11^. Thus, locally tailored research is important to understand the reason behind the higher incidence of GBC and identify as to whether any preventive measures could be adopted.

According to the American Cancer Society, only about 1 in 5 GBC cases will be discovered when the disease is still localized to the gallbladder^12^. The remaining cases are diagnosed when the malignancy has spread outside of the gallbladder, which drastically limits the available options for curative treatment and lowers overall survival. In line with the aforementioned study, 90% of those who underwent curative intent surgery presented to us with an advanced stage of tumor (T3, N1). Unfortunately, around 10% of our patients in the study were young (<40 years) onset GBC. They were predominantly metastatic, advanced staged with less than a year of survival even after extended resections. Seventy percent (70%) of the patients had associated gallstones. This recommends for proper GBC cancer surveillance, especially considering all benign cases of cholecystectomies with a histopathological examination and addressing the associated risk factors like gallstones in context to Nepal.

The majority of GBC patients who present with jaundice will have disseminated disease even if it is not detectable on preoperative work-up or operative exploration. The en-bloc resection of the CHD/CBD, which is frequently required in these patients, is difficult and associated with positive (R1) margin status in 40% of patients^13^,^14^. Despite anecdotal reports of longer postoperative survival in GBC patients presenting with the rare combination of jaundice without nodal involvement^15^, even in patients with a negative (R0) margin, the median length of disease-free survival in preoperatively jaundiced patients is only 6 months^16^. Based on these data, preoperative jaundice is considered a relative contraindication to radical resection of the GBC. In contrast to the aforementioned, 56% of our patients presented with jaundice. Out of 30% of patients who underwent curative intent surgery, a median overall survival of 28 months after a median follow-up of 42 months (12–64 months) was reported. Our published study has shown, curative resection was possible in 9% (5/56) patients in GBC with obstructive jaundice^17^. Thus, with reference to our study, GBC with jaundice can no longer be considered as a contraindication to resection, albeit with a low rate.

Likewise, long-term survival after radical resections that included major hepatectomy, CHD/CBD and/or vascular resection or reconstruction has been anecdotally reported^14^, but these radical resections have not been associated with longer disease-free or overall survival on a population basis. Instead, they are associated with increased morbidity and mortality. Radical resections of locally advanced primary tumors should, therefore, be performed only in medically fit patients after multidisciplinary discussion. Although R0 resection for GBC is associated with longer survival, tumor biology and stage, rather than the extent of resection, are the most important predictors of survival after surgery^13^.

The median lymph node yield in the present study was only four, which is low compared to the current guideline recommendation of six. It was multifactorial. First, the actual number of nodes present in the hilar region may be few. Only the current eighth edition American Joint Committee on Cancer after 2018 recommended removal of a minimum of six lymph nodes for adequate staging of GBC^18^. Furthermore, there may be a lack of awareness amongst pathologists for this number of LNs. If we see the world’s largest published series, only 30% of the resected specimen achieve LNs more than or equal to three. After 2018, our LNs yield has increased, and we are more conscious on the number of LNs for adequate staging of GBC^18^.

In our study, perioperative morbidity and mortality were 23 and 3%, respectively. The overall survival of the entire group was 9 months. Out of 30 patients who underwent curative intent surgery, 20 (66.67%) had R0 resection. A median overall survival of 28 months after a median follow-up of 42 months (12–64 months) was reported. This adequately supports the notion that radical resection for GBC renders a good prognosis and prolonged overall survival. According to our study, only 30% of those presenting to us, undergo curative intent surgery. Furthermore, only 12 (40%) of them have completed adjuvant chemotherapy. This low adjuvant therapy completion is because of lack of health insurance coverage, financial burden, geographical status, and poverty.

In our study, 7% (*n*=7) had incidental GBC, quite a high incidence. Out of them, 57.1% (*n*=4) underwent curative intent surgery with a good prognosis. The remaining had metastatic disease due to port site metastases and delayed presentation. GBC is suspected preoperatively in only 30% of all patients. The remaining 70% of cases are diagnosed using postoperative incidental findings by a pathologist. Incidental GBC is high by 2–3 times compared to total GBC. In a study by Poudel and Shah^19^ incidence of IGBC in cholecystectomies specimens for benign disease is 1.67%. They recommended that routine histopathology of cholecystectomy specimen should be sent for early diagnosis and to improve the survival of patient with gallbladder cancer. Hence, the gallbladder should be evaluated histo-pathologically in all patients after laparoscopic cholecystectomy, as these are the group of patients with early-stage disease with better survival.

Few published studies have focused on the patterns and timing of recurrence after resection of GBC. Despite curative intent resection, up to 66% of resected patients develop recurrence, mostly within 2 years of surgery, often at the distant site^20^. In line to the aforementioned, out of 30 patients who underwent curative intent surgery, 46.6% (*n*=14) had recurrence in the present study. Three patients had a local recurrence only, three patients had metastatic disease and a local recurrence, and eight patients had metastatic disease only. This high recurrence was because of low R0 resection, a lack of completion of adjuvant chemotherapy, and advanced stage disease.

Our study has some limitations. The study is limited by the small sample size of patients with GBC undergoing surgery with a curative intent. Also, this is a single centered study. Otherwise, more robust evidence could have been delivered with a multi-center study. However, the best part of the study is that, it is one of the preliminary and prospective studies with 3 years follow-up from our part. A larger sample size, government health insurance coverage for overall treatment and multi-institutional involvement, henceforth, may provide a better insight to the incidence and prognosis of GBC.

## Conclusion

The study demonstrates that in GBC, provided multimodality approach, aggressive surgical resection with curative intent is feasible in only one-third of patients with an acceptable operative morbidity/mortality. Surgery provides a survival benefit in curative and palliative group of patients. However, the majority (70%) of GBC patients are at advanced stage disease and unresectable at the time of presentation. Neoadjuvant therapy, health insurance, ultrasound surveillance for GB stone, and prophylactic cholecystectomy may result in a better outcome of these patients.

## Ethics approval

Ethical approval for this study was obtained from Institutional Review Board approval (10.082), B.P. Koirala Institute of Health Sciences, Dharan, Nepal.

## Consent to participate

Informed consent was obtained from all individual participants included in the study.

## Consent to publish

The authors affirm that human research participants provided informed consent for publication of the findings in this research.

## Sources of funding

The authors declare that no funds, grants, or other support were received during the preparation of this manuscript.

## Author contribution

All authors contributed to the study conception and design. Material preparation, data collection and analysis were performed by Narendra Pandit, Durga Neupane, Dinesh Nalbo, Sameer Bhattarai, Kunal Bikram Deo, Lokesh Shekher Jaiswal and Shailesh Adhikary. The first draft of the manuscript was written by Narendra Pandit and Durga Neupane and all authors commented on previous versions of the manuscript. All authors read and approved the final manuscript.

## Conflicts of interests disclosure

The authors have no relevant financial or non-financial interests to disclose.

## Research registration unique identification number (UIN)

1. Name of the registry: Not available.
2. Unique Identifying number or registration ID: Not available.
3. Hyperlink to your specific registration (must be publicly accessible and will be checked): Not available.

## Guarantor

Narendra Pandit.

## Data availability statement

The datasets generated during and/or analyzed during the current study are available from the corresponding author on reasonable request.

## Provenance and peer review

Not commissioned, externally peer reviewed.
